# A single-nuclei RNA sequencing study of Mendelian and sporadic AD in the human brain

**DOI:** 10.1186/s13195-019-0524-x

**Published:** 2019-08-09

**Authors:** Jorge L. Del-Aguila, Zeran Li, Umber Dube, Kathie A. Mihindukulasuriya, John P. Budde, Maria Victoria Fernandez, Laura Ibanez, Joseph Bradley, Fengxian Wang, Kristy Bergmann, Richard Davenport, John C. Morris, David M. Holtzman, Richard J. Perrin, Bruno A. Benitez, Joseph Dougherty, Carlos Cruchaga, Oscar Harari

**Affiliations:** 1 Department of Psychiatry, Washington University School of Medicine, Campus Box 8134, 425 S. Euclid Ave, BJC Institute of Health, Office: 9607, St. Louis, MO 63110 USA; 2 Hope Center for Neurological Disorders, Washington University School of Medicine, St. Louis, MO USA; 3 Knight Alzheimer’s Disease Research Center, Washington University School of Medicine, St. Louis, MO USA; 4 NeuroGenomics and Informatics, Department of Psychiatry, Washington University, St. Louis, MO USA; 5 Department of Neurology, Washington University School of Medicine, St. Louis, MO USA; 6 Department of Pathology and Immunology, Washington University School of Medicine, St. Louis, MO USA; 7 Department of Genetics, Washington University School of Medicine, St. Louis, MO USA

**Keywords:** *PSEN1*, Single-nuclei RNA-seq, Alzheimer’s disease, Web-based brain molecular atlas

## Abstract

**Background:**

Alzheimer’s disease (AD) is the most common form of dementia. This neurodegenerative disorder is associated with neuronal death and gliosis heavily impacting the cerebral cortex. AD has a substantial but heterogeneous genetic component, presenting both Mendelian and complex genetic architectures. Using bulk RNA-seq from the parietal lobes and deconvolution methods, we previously reported that brains exhibiting different AD genetic architecture exhibit different cellular proportions. Here, we sought to directly investigate AD brain changes in cell proportion and gene expression using single-cell resolution.

**Methods:**

We generated unsorted single-nuclei RNA sequencing data from brain tissue. We leveraged the tissue donated from a carrier of a Mendelian genetic mutation, *PSEN1 p.A79V*, and two family members who suffer from sporadic AD, but do not carry any autosomal mutations. We evaluated alternative alignment approaches to maximize the titer of reads, genes, and cells with high quality. In addition, we employed distinct clustering strategies to determine the best approach to identify cell clusters that reveal neuronal and glial cell types and avoid artifacts such as sample and batch effects. We propose an approach to cluster cells that reduces biases and enable further analyses.

**Results:**

We identified distinct types of neurons, both excitatory and inhibitory, and glial cells, including astrocytes, oligodendrocytes, and microglia, among others. In particular, we identified a reduced proportion of excitatory neurons in the Mendelian mutation carrier, but a similar distribution of inhibitory neurons. Furthermore, we investigated whether single-nuclei RNA-seq from the human brains recapitulate the expression profile of disease-associated microglia (DAM) discovered in mouse models. We also determined that when analyzing human single-nuclei data, it is critical to control for biases introduced by donor-specific expression profiles.

**Conclusion:**

We propose a collection of best practices to generate a highly detailed molecular cell atlas of highly informative frozen tissue stored in brain banks. Importantly, we have developed a new web application to make this unique single-nuclei molecular atlas publicly available.

**Electronic supplementary material:**

The online version of this article (10.1186/s13195-019-0524-x) contains supplementary material, which is available to authorized users.

## Background

Alzheimer’s disease (AD) is a neurodegenerative disorder characterized by the presence of amyloid Aβ plaques and neurofibrillary tangles (hyperphosphorylated tau deposits) in the brain [1]. AD is also associated with neuronal death and gliosis specifically in the cerebral cortex. AD has a substantial but heterogeneous genetic component. While carriers of mutations in the *amyloid-beta precursor protein* (*APP*) and *Presenilin* genes (*PSEN1* and *PSEN2*) [2, 3] show Mendelian inheritance patterns, the majority of the AD cases (90–95%) present a complex genetic architecture (sporadic AD), with many genetic factors contributing to risk. Recently, we studied the cellular population structure of AD brains of carriers of Mendelian mutations in *APP*, *PSEN1*, and *PSEN2*, and sporadic ADs and compared them to neuropathology-free controls [4]. To do so, we generated bulk RNA-seq from the parietal lobe and analyzed it using an optimized digital deconvolution method [4] to infer broad proportions of neurons, astrocytes, oligodendrocytes, and microglia. Importantly, we identified that the brains of the carriers of Mendelian mutations have a specific distribution of neurons that differs from that of sporadic AD [4]. However, bulk RNA-seq and deconvolution approaches do not provide a detailed context of expression profiles at the cellular level, which hampers the identification of which neuronal subtypes [5] are the most vulnerable to the AD pathogenesis.

Single-cell RNA-seq (scRNA-seq) provides the opportunity to generate the detailed transcriptomic cell profiling required to address the drawbacks of bulk RNA-seq [6–8]. However, performing scRNA-seq requires cell re-suspension, and library preparation needs to be done from fresh tissue. This requirement prohibits the study of highly informative frozen human brain tissue stored in brain banks. Single-nuclei RNA-seq (snuclRNA-seq) is an alternative to this methodology. Studies of mouse neural progenitor cells show that only small differences are present between total cellular and nuclear RNA profiles [9]. We sought to investigate the feasibility of generating and employing unsorted snuclRNA-seq to analyze banked brains from related individuals, which exhibit Mendelian or sporadic genetic architectures of AD. We hypothesize that cell-type expression profiling data from these brains will provide a unique resource to (i) obtain a highly detailed map of cell composition in human brains of AD, (ii) determine cellular population structure changes in common and specific to Mendelian and sporadic AD, and (iii) characterize transcriptomic profile alterations in AD within each defined cell type.

We discovered that analyzing snuclRNA-seq from the human brains has its own challenges, as most of the current tools and pipelines are set up to analyze scRNA-seq obtained from fresh tissue. When applying these to snculRNA-seq, we encountered artifacts and biases that constrained downstream analyses. Thus, we propose an approach to extend scRNA-seq analyzing methodology to snuclRNA-seq to identify clusters of nuclei that show trustworthy expression profiles, and which resemble distinct subtypes of neurons and glial cells, including oligodendrocytes, microglia, and astrocytes, among others. In addition, these clusters have an even (fair) representation of the nuclei from all of the donors, a property required by many single-nuclei downstream analyses. We then employed this approach to process the snuclRNA-seq from human frozen brains and analyze the distribution of neuronal subtypes in the brains with Mendelian and complex genetic architectures of AD.

Recently, a novel type of microglia has been proposed to be associated with neurodegeneration [10]. The disease-associated microglia (DAM) was identified generating scRNA-seq from sorted microglial cells from mouse models [10] and then validated using immunohistochemical staining of human brain tissue to demonstrate the colocalization of Aβ particles with a protein marker of DAM [10]. Here, we analyze whether the expression profile of human microglia detected by snuclRNA-seq recapitulate the DAM expression signature detected in mouse models.

Finally, our findings indicate that snuclRNA-seq provides a valuable resource that can improve our understanding of the sequence of regulatory events that control cell fate leading to AD. Therefore, we are making available the nucleus-specific expression profile of the brains we analyzed through an interactive web-based application (http://ngi.pub/snuclRNA-seq) to provide full access to the research community to this resource. This is the first single-nuclei molecular atlas of AD brains carrying pathological mutations in *PSEN1* and related sporadic AD. We hope that this high-quality data will help elucidate and validate novel biological insights into AD and contribute to early diagnosis and therapeutic intervention.

## Methods

### Samples

The Neuropathology Core of the Knight-Alzheimer’s Disease Research Center (Knight-ADRC) provided the parietal lobe tissue from the postmortem brains for each sample. These samples were obtained with informed consent for research use and were approved by the institutional review board of Washington University in St. Louis. AD neuropathological changes were assessed according to the criteria of the National Institute on Aging-Alzheimer’s Association (NIA-AA). One AD patient is a carrier of the *PSEN1* p.A79V pathogenic mutation, while the two relatives do not carry any autosomal mutations (sporadic AD). The three donors are females with European-American ancestry. Additional information including the clinical dementia rating (CDR), *APOE* genotype, postmortem interval (PMI), age at onset, and age at death are detailed in Table 1.

**Table 1:** Demographic characteristics of the samples

	Sample1	Sample2	Sample3
PSEN1 mutation	Non-carrier	Non-carrier	Ala79Val
Gender	Female	Female	Female
Age on set (years)	87	81	76
Age at death (years)	89	82	89
CDR	1	3	3
APOE	ɛ3/ɛ4	ɛ3/ɛ3	ɛ3/ɛ4
PMI	9	17.2	8
Freezer time^&^ (years)	11	22	24
Braak amyloid beta*	C	NA	C
Neuropathology^&^	AD	AD	AD

^&^Freezer time: the years that elapsed since the death to snuclRNA-seq library preparation. *C: Stage C (deposition of amyloid in isocortical areas). *AD* Alzheimer’s disease, *NA* no data

### Nuclei extraction and library preparation

From the fresh frozen human parietal lobes, approximately 500 mg of tissue was cut and weighed on dry ice using sterile disposable scalpels. The parietal tissue was homogenized in ice-cold homogenization buffer (0.25 M sucrose, 150 mM KCl, 5 mM MgCl2, 20 mM Tricine-KOH pH 7.8, 0.15 mM spermine, 0.5 mM spermidine, EDTA-free protease inhibitor, and recombinant RNase inhibitors) with a glass-on-glass dounce homogenizer, 10 strokes with the A pestle, followed by 10 strokes of the B pestle. Homogenates were centrifuged for 5 min at 500×*g*, at 4 °C, to pellet the nuclear fraction. The nuclear fraction was mixed with an equal volume of 50% iodixanol and added on top of a 35% iodixanol solution for 30 min at 10,000×*g*, at 4 °C. After the removal of the myelin layer from the top of the gradient, the nuclei were collected from the 30–35% iodixanol interface. The nuclei were resuspended in nuclei wash and resuspension buffer (1.0% bovine serum albumin and recombinant RNase inhibitors in phosphate-buffered saline) and pelleted for 5 min at 500 × Gs, 4 °C. The nuclei were passed through a 40-μM cell strainer to remove cell debris and large clumps. Nuclei concentration was manually determined using DAPI counterstaining and hemocytometer. Nuclei concentration was adjusted to 1200 nuclei/μL and followed immediately by the 10X Genomics® Single Cell Protocol. We generated snuclRNA-seq libraries using the 10X Chromium V(D)J 5` chemistry for 10,000 cells per sample and sequenced 50,000 reads per cell from the 3 frozen human parietal lobes.

### Sequencing alignment and data for secondary analysis

The CellRanger (v2.1.1 10XGenomics) software was employed to align the sequences and quantify gene expression. By default, this software quantifies the expression for mature messenger RNA (mRNA) by counting reads aligned to exons as annotated in the human genome build GRCh38. However, the snuclRNA-seq profiles nuclear precursor mRNA (pre-mRNA), which includes transcripts that have not completed splicing to remove introns. To capture all the information in the pre-mRNA, we aligned the reads to a custom “pre-mRNA” reference that was generated as described by 10X Genomics technical manual [6]. In this way, the intronic reads from pre-mRNA are included in the final gene expression counts. We aligned and quantified the gene expression using both mature and pre-mRNA references, and these reads were further cleaned QCed and compared.

### Single-nuclei RNA-seq cleaning

The gene expression matrices from all samples were combined in R independently for further processing using the Seurat (version 2.20, 2.30) pipeline. We processed the gene expression quantified for the pre-mRNA and mRNA references in parallel. We removed most of the mitochondrial genes (< 0.1%) and then kept the genes expressed in three or more cells. We discarded cells with less than 1800 or more than 8000 genes expressed. To exclude multiplets, when a single droplet includes two or more nuclei, the top 0.5% of the distribution of nUMI (total number of unique molecular identifiers detected in each cell) were removed. We also studied whether the number of genes expressed per nuclei followed a multi-modal distribution, under the assumption that glial cells could express a lower number of genes than neurons [11]. This analysis was performed by the R package mixtools [12]. We normalized the data employing the *LogNormalize* function, which normalizes the gene expression measurements for each cell by the total expression; scales by a factor equal to the median counts of all genes and log-transforms the expression. Data regression was performed using the *ScaleData* function with nUMI, percent mitochondrial reads, and sample origin as confounding factors.

### Selection of genes for cell clustering

The snuclRNA-seq expression data is highly dimensional, as thousands of nuclei are ascertained transcriptome-wide for each brain. However, as single-cell methods are lossy, the quantification of snuclRNA-seq produces sparse data matrices, and the expression of each gene is not detected for each nucleus. The structure of the data complicates the clustering of the nuclei into distinct cell types, and conventional similarity distances, such as Euclidean distance, have been claimed to be less reliable as the dimensions of the feature space increase [13]. However, only hundreds of genes are required to discriminate cell types. The selection of a representative set of genes to cluster the nuclei is a key step in the processing of data, in which only the informative genes are filtered, in such a way that clustering of nuclei is computationally feasible. Given the importance of this processing step, we evaluated alternative approaches, described below.

#### Classic Gene Set from pooled subjects

The Classic Gene Set (CGS) method is the approach most commonly employed to select the most variable genes in scRNA-seq studies [14, 15]. The Seurat *FindVariableGenes* function performs this selection. We executed this method by using the default values and cutoffs (x.low.cutoff = 0.0125, x.high.cutoff = 3, y.cutoff = 0.8) and selected the most variable genes from the single nuclei expression from the three brains. We obtained 2360 genes that were used to calculate 100 principal components (PCs). Then, we identified the optimal number of PCs for downstream analysis using heuristics, PC elbow plot, and JackStraw statistical tests. The *JackStraw* function randomly permutes a subset of data and calculates projected PC scores for these genes. This analysis indicated that the first 65 PCs were sufficient for clustering the nuclei.

#### Hicat Gene Markers

This method proposed by Taasic et al. [16] employs known gene markers of cell types to generate an initial partition of the cells into broad cell clusters. Later on, this initial partition can be subclustered to identify cell subtypes. For our analysis, we employed all the nuclei that passed QC, using 118 known gene markers, that we collected from the literature (Additional file 1: Table S1) [5, 11, 17–19]. We calculated the first 100 PCs from the expression of these marker genes, as quantified using the pre-mRNA annotation (Seurat software). We selected the number of PCs using the same methodology as described in the “Classic Gene Set from pooled subjects” section and selected the first 55 PCs for downstream analysis.

#### Consensus Gene Set

The approach we propose, the Consensus Gene Set (ConGen), controls for biases and obtains clusters with even representation of all samples. We applied the Seurat *FindVariableGenes* with default selections and cutoffs values (x.low.cutoff = 0.0125, x.high.cutoff = 3, y.cutoff = 0.8) for each library sample and identified a set of highly variable genes for each brain sample whose expression was quantified using pre-mRNA annotation. The number of highly variable genes is 2447, 2354, and 1972 for Sample1, Sample2, and Sample3, respectively. Then, we identified the common set of genes that were highly variable among the samples using the R function intersection (*N* = 1434). These common genes were used to calculate 100 PCs from all of the samples (Seurat package). We selected the number of PCs using the same methodology described in the “Classic Gene Set from pooled subjects” section and employed the first 25 PCs for downstream analysis.

### Cell/nuclei clustering

The goal of this process is to group the nuclei based on the similarities between their expression profiles. We used the Seurat function *FindClusters* to identify the clusters with a resolution parameter 0.6 and employed the *TSNEPlot* function to generate a visual representation of the clusters using T-distributed Stochastic Neighbor Embedding (tSNE). In addition, we corrected for dropout events that lead to an exceedingly sparse depiction of the single-cell transcriptome. Instead of removing genes containing missing values, which restricts the analysis to only highly expressed genes, we imputed missing gene expression values using *scImpute* [20]. After imputing gene expression, we clustered the nuclei again. Finally, we learned similarity relationships among the expression profile of the cells included in the clusters by using the function *BuildClusterTree*.

### Coincidence analysis

To compare the results of distinct clustering approaches and identify whether three different approaches were producing (dis)similar clusters, we implemented a coincidence analysis. We performed an exhaustive comparison of each cluster identified by one processing and clustering approach to all of the clusters identified by a second approach. In this way, we can determine how the cells are reorganized among different clusters or, in contrast, to identify if cells are grouped coincidently together by distinct approaches. To do so, we calculate the normalized pointwise mutual information (pmi):

$$ \mathrm{pmi}\left(x,y\right)=\log \frac{p\left(x,y\right)}{p(x)p(y)} $$

which quantifies the discrepancy between the probability of joint distribution of two clusters and their individual distributions. In addition, we calculated the Jaccard index, or similarity coefficient, which is defined as:

$$ \mathrm{Jaccard}\left(x,y\right)=\frac{\mid x\cap y\mid }{\mid x\cup y\mid } $$

The Jaccard index evaluates the extent of the intersection of the nuclei in common between two clusters, corrected by the extent of the union of the nuclei assigned to the two clusters. To provide a global overview of the clusters’ coincidence, we plotted the pointwise comparisons and we represented the *pmi* by color and the *Jaccard index* by the size of the point (Online Resource: https://github.com/NeuroGenomicsAndInformatics/snuclRNA-seq/tree/master/tools).

### Entropy as a measure to evaluate donor evenness and biases in clustered nuclei

Having an even representation of the nuclei from distinct samples in a cluster is critical to perform unbiased comparisons. Otherwise, clusters of nuclei with overrepresentation or underrepresentation of samples precludes many downstream analyses. We employed Shannon’s information theory entropy [21] as a quantitative measure to evaluate how even or biased the distribution of samples among the nuclei of any given cluster is. Specifically, the entropy of a cluster is calculated as:

$$ \hat{H}=-\sum p(i){\log}_2\left(p(i)\right) $$

where *p*(*i*) is the probability of the nuclei belonging to the subject *i*. The entropy tends to 0 when all of the cells from a cluster belong to a single subject and is maximized when the distribution of subjects is perfectly uniform which is 1.58 for three samples (Online Resources: https://github.com/NeuroGenomicsAndInformatics/snuclRNA-seq/tree/master/tools).

### Cluster annotation

We determined the brain cell types in each of the cluster by evaluating the expression of maker genes for neurons, astrocytes, oligodendrocytes, microglia, oligodendrocyte precursor cells, and endothelial cells, usually employed in the literature [5, 11, 17–19] (Additional file 1: Table S2 and Table S3). We used the *DotPlot* function from the Seurat package to visualize the average expression of genes related to specific cell types. To determine the homogeny of brain samples analyzed, we also evaluated the expression of marker genes tagging distinct pyramidal layers for the excitatory neurons. We also looked for excitatory and inhibitory neurons [5].

### Pseudo-temporal trajectories of microglia expression profiles

The gene expression for the microglia was analyzed using the R package TSCAN (version 1.7.0) to infer a pseudo-temporal path [22]. This method orders cells based on the transition of their transcriptomes, by initially clustering cells then identifying similarity relationships among the clusters, inferring a minimal spanning tree (MST), projecting cells onto tree edges and finally using generalized additive models to ascertain the functional relationship between the pseudo-time and gene expression. The raw expression data from microglial cells was loaded and pre-processed (TSCAN function *preprocess*) using default parameters. We then executed the *exprmclust* function with default parameters to learn the optimal number of clusters for microglia. Then, we employed the *tscan* function in order to identify pseudo-time trajectories of the cells. Finally, we used the processed expression data and the pseudo-order to identify the genes associated with this ordering, using the *difftest* function.

### Single-nuclei gene expression browser

We developed a web application to provide public access to the single-nuclei transcriptomic atlas. All of the snuclRNA-seq data that we generated and processed can be queried and visualized using a custom browser that we have developed using the R Shiny framework. This browser provides an input panel to query individual genes and additional parameters to customize the visualization. For each gene, the browser provides graphical information organized in three panels: (i) The tSNSE projection of the nuclei is represented and colored by the clusters. We identified and annotated these clusters using Seurat *TSNEPlot* function. (ii) A gene expression level for each nucleus is graphically represented using the same tSNE representation. The nuclei are colored according to the expression level of the queried gene. We represent the nuclei highly expressing the target gene in purple and the nuclei with lower expression in gray (Seurat *FeaturePlot* function). (iii) We show the cell type-specific differential expression (log fold change, *p* value, and adjusted *p* value) of queried genes for neurons, astrocytes, microglia, oligodendrocyte, oligodendrocyte precursor cells (OPC), and endothelial cells that was pre-computed beforehand. We first grouped the clusters by cell type (Seurat *BuildClusterTree* function). As a result, we obtained a unique cluster that includes all of the neurons, while conserving clusters for each of the remaining cell types. Thus, the nuclei are partitioned into a total of six clusters. Then, we calculated the differential expression for each gene among these six clusters using the Seurat *FindMarkers* function. The statistical significance was calculated using the non-parametric Wilcoxon rank-sum test to determine those genes that had a log fold change > 0.01. In this way, we generated a broader comparison of the transcriptome. The adjusted *p* value is adjusted using a Bonferroni multiple test correction. All of the source code for the snuclRNA-seq explorer and the precomputation of the differential expression is available in the GitHub repository (https://github.com/NeuroGenomicsAndInformatics/snuclRNA-seq/).

### Bulk RNA-seq processing

In parallel, we generated bulk RNA-seq data for the three brains that we analyzed and followed the same QC and processing as we reported previously [4]. These data were mapped to mRNA reference for the human genome build GRCh38.

### Ascertaining snuclRNA-seq quality

To control for the quality of the processed transcriptomic snuclRNA-seq, we compared the gene expression values from both alignments for each subject and, as a group, to their gene expression values from their bulk RNA-seq.

## Results

### Study design

We collected snuclRNA-seq data from three European-American female donors. We employed two different references for alignment, pre-mRNA and mRNA, to quantify the gene expression for each sample donor. Each sample was accompanied by the processing of a matching bulk RNA-seq sample using mRNA as a reference. These steps allowed us to estimate how well snuclRNA-seq data can recapitulate the RNA-seq results from bulk samples. The gene expression matrices from all samples were combined for further processing. Three different approaches were tested to select the variable genes that were used to determine the principal components for the nuclei clustering. We processed each approach in parallel. We tested the evenness of each cluster for each approach as well as critical parameters such as resolution and coincident analysis. The approach with the best results was then imputed and used for further analyses including the pseudo-time trajectories of the cells and disease-associated microglia association. Finally, we prepared a browser using the R Shiny framework to visualize the snuclRNA-seq data that we produced.

### Clinical and demographic characteristics

The complexity and uniqueness of the cell types in the different regions and layers in the human brain were previously described [5, 23–28]; however, the sample quality is diminished in postmortem samples. To obtain highly detailed maps of cell composition in AD brains with distinct genetic architecture and to characterize their transcriptomic profiles at a cellular level, we analyzed snuclRNA-seq from the parietal lobe [29–32], for three subjects (Additional file 1: Figure S1). All three donors were European-American females, with an age of death ranging from 82 to 89 years and were members of the same family. One of the donors, Sample3, was a carrier of the *PSEN1* p.A79V (ADAD) while the other two donors, Sample1 and Sample2, presented a complex genetic architecture of AD. Neuropathology showed definitive AD for all the samples. The Braak amyloid stage for Sample1 and Sample3 is C (deposition of amyloid in isocortical areas); no data was recorded for Sample2. Sample collection or postmortem interval (PMI) ranged between 8 and 17.2 h (Table 1). As previously reported [5, 33, 34], we did not observe any effect of the PMI of the samples and quality of snuclRNA-seq. Neither did we observe any effect of the lapse of time the samples were frozen until we generated the data (freezer time) and quality of the data.

### Generation and quality evaluation of human brain tissue single-nuclei RNA-seq

We generated snuclRNA-seq libraries using the 10× Chromium for 10,000 cells per sample and sequenced 50,000 reads per cell from 3 frozen human parietal lobes. We employed 2 different alignment libraries for the annotation of the genome, pre-mRNA and mRNA, and quantified gene expression using both (see the “Methods” section). We observed that when we aligned the reads to pre-mRNA, the number of reads, genes, and cells increases significantly. For example, when using pre-mRNA as a reference, we observed a 36% increase in the number of nuclei (26,331 vs 19,302; *χ*^2^ test, *p* ≤ 2.2 × 10^−16^, Table 2), without affecting the number of identified transcripts (28,428 vs 25,465 counts). We also observed a 118.8% increase in the number of the median UMI counts per nuclei (*t* test, *p* = 1.5 × 10^−2^) and a 45.1% for the median genes per nuclei (*t* test, *p* = 5.0 × 10^−3^).

**Table 2:** Summary statistics after quality control

	Sample1	Sample2	Sample3	All
Alignment to precursor RNA (pre-RNA)				
Number of nuclei	5663	7147	13,521	26,331
Median UMI counts per nuclei	11,560	14,444	6543	9262
Median genes per nuclei	4006	5064	2852	3642
Total genes detected	28,428	28,428	28,428	28,428
Alignment to mature-RNA				
Number of nuclei	4493	7197	7612	19,302
Median UMI counts per nuclei	3960	7162	2464	4234
Median genes per nuclei	2355	3851	1579	2510
Total genes detected	25,469	25,469	25,469	25,469

**Fig. 1:**
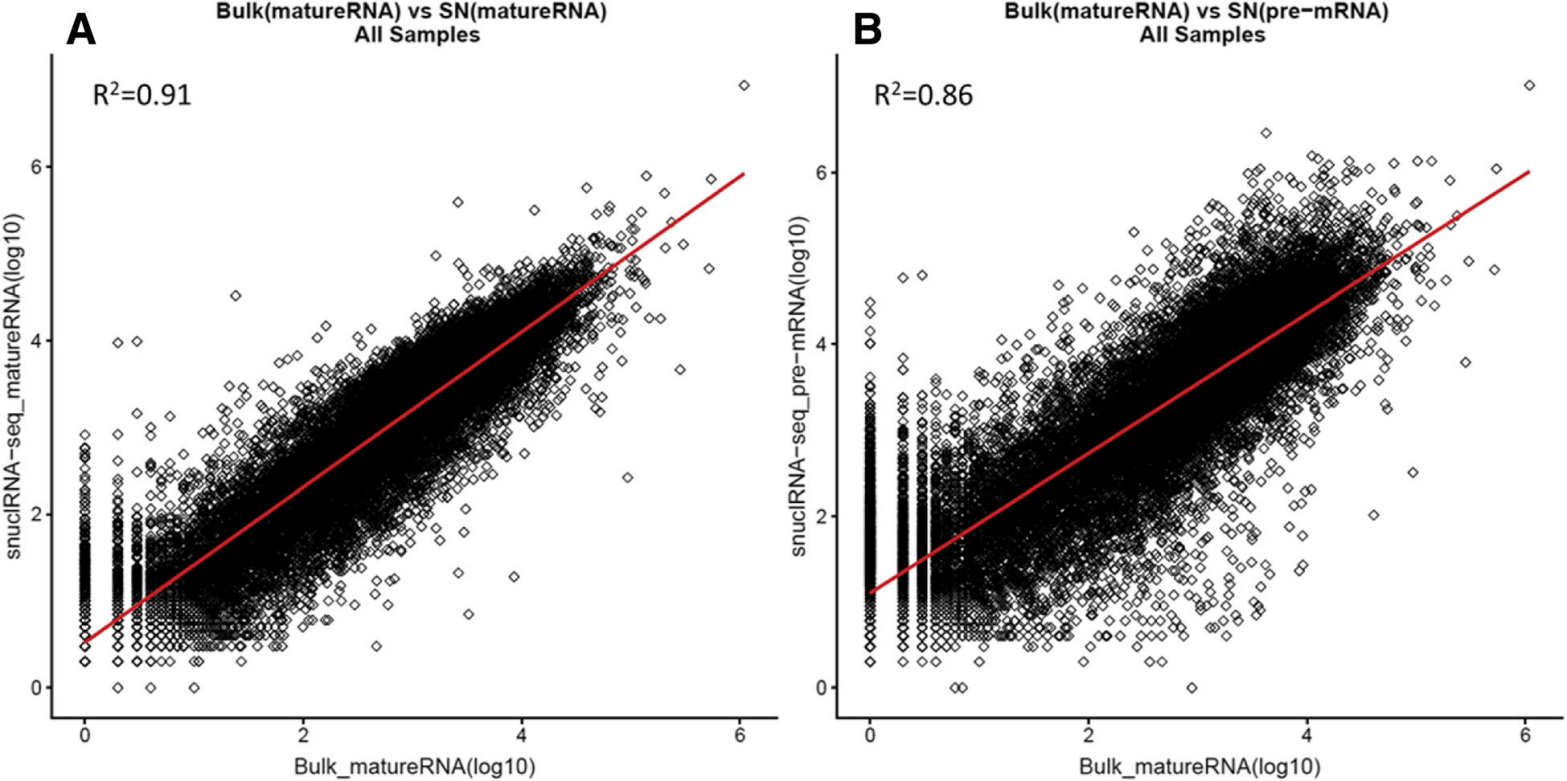
Correlation between bulk RNA-seq and single-nuclei RNA-seq aligned using the pre-mRNA and mRNA annotation references. Along the *X*-axis, we show the gene expression values obtained from the bulk RNA-seq, and along the *Y*-axis, the single-nuclei expression, which was analyzed as bulk RNA-seq. **a** Bulk RNA-seq vs snuclRNA-seq aligned with mRNA (see the “Methods” section). **b** Bulk RNA-seq vs snuclRNA-seq aligned with pre-mRNA

To further evaluate the quality of snuclRNA-seq data, we compared the gene expression values from the two alignments “pre-mRNA” and “mRNA” in snuclRNA-seq to their gene expression values from their parallel bulk RNA-seq aligned to the mRNA reference data (Table 3). We observed a Pearson correlation of *r*^2^ = 91% between snuclRNA-seq with mRNA reference and bulk RNA-seq (Fig. 1a). On the other hand, the Pearson correlation of snuclRNA-seq with pre-mRNA reference against bulk RNA-seq was *r*^2^ = 86% (Fig. 1B). These results may suggest that aligning to mRNA is a better approach, but it is important to note that the alignment to pre-mRNA increased the number of nuclei, median UMI counts per nuclei, and median genes per nuclei significantly. In addition, the correlation we identified for the pre-mRNA is in line with the values previously reported for single-cell RNA-seq [35].

**Table 3:** Correlation analysis between bulk RNA-seq and snuclRNA-seq after QC

	Reference alignment	Bulk RNA-seq mature-RNA
snRNA-seq	Pre-RNA	0.86
	Mature RNA	0.91

All values are Pearson coefficient

These results indicated that the inclusion of introns for quantifying the gene expression of snuclRNA-seq data provides a better description of the nuclei-specific expression profile, while conserving a correlation to the expression of bulk RNA-seq, which validates the accuracy of this data.

### CGS approaches to cluster single nuclei from human postmortem brains

**Fig. 2:**
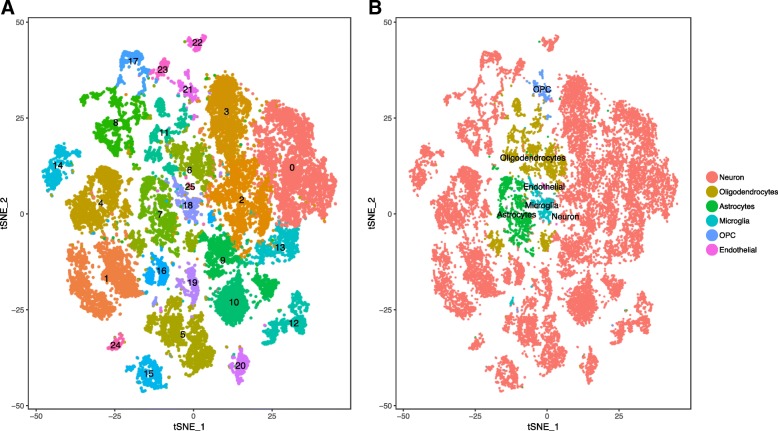
TSNE plots for the CGS dimensional reduction approach. TSNE plots depicting 26,331 nuclei. **a** The nuclei are colored to represent the 25 CGS clusters. **b** The clusters are annotated to represent the cell types (neuron, ologodendrocytes, astrocytes, microglia, OPC, and endothelial)

We initially sought to identify different cell types in the brain samples by a CGS approach that performs an unsupervised graph-based clustering [36] to identify the groups of cells with similar expression profiles. This approach detected 25 clusters using 2360 highly variable genes using the default resolution of 0.6 (Additional file 1: Table S4, Fig. 2a). The clusters were annotated in six cell types (Fig. 2b, Additional file 1: Figure S2); however, we noticed that many clusters included cells that were not evenly distributed among the three donors, but instead showed an overrepresentation or an underrepresentation of donors. Specifically, Sample2 is overrepresented in cluster 0 and underrepresented in clusters 2 and 3. Similarly, Sample3 is overrepresented in clusters 7 and 10 (Additional file 1: Table S4, Fig. 2b). We employed Shannon’s entropy to formally quantify the evenness of cell distribution among donors (see the “Methods” section). This a metric that is maximized when the probability distribution is uniform. For the three brains we analyzed, its values ranged from 0 to 1.58. An even distribution of the three brain samples in a cluster would result in entropy > 1.2. Lower values to this cutoff would be expected for a cluster with a 65% of overrepresentation of cells from a single donor or an underrepresentation of 10%. We observed that for clusters 0, 2, 3, 7, and 10, the entropy < 1.2 (Additional file 1: Table S4). Note that these clusters that exhibit uneven distribution of subjects included 9781 nuclei, which represents 37.1% of the nuclei that passed QC. These clusters would not allow us to perform downstream comparisons on a relatively large proportion of the cells we sequenced, as many of these analyses require fair sampling of cells from the distinct donors.

We observed that generating a distinct number of clusters (by sampling the parameter resolution with values between 0.2 and 1.2) conserved the overall (nested) structure of the clusters. Higher resolution values tended to partition specific clusters, although cells were not reorganized in clusters with an even distribution of samples. When we employed lower resolution values (e.g., 0.2), the clusters started to show higher entropy values, as they grouped cells from all of the three donors. However, these clusters did not present cell type-specific expression profiles. These results indicate that when analyzing snuclRNA-seq from human-related brains, this approach produces some biases that constrain the comparisons for a large number of cells. This bias could be addressed by the reduction of the resolution in the analysis, but the expression profile for these new clusters is not specific.

### The Hicat Gene Markers approach does not generate a trustworthy cell type-specific expression profile

Next, we evaluated whether clustering nuclei based on genes usually employed as cell type marker would produce cell type-specific clusters. This approach, called “Hicat Gene Markers” [16], has been previously employed to analyze cell sorting (FACS) neurons. Using cell type markers (see the “Methods” section; Additional file 1: Table S1), we clustered the nuclei into 23 bins (resolution 0.6 Additional file 1: Figure S3). We observed that the nuclei were better distributed among the samples, and the entropy values were closer to 1.58 (Additional file 1: Table S5) which is the maximum value that indicates perfectly even distribution. However, annotation of the clusters indicated that this method is not grouping homogenous cell types, as these clusters did not present cell type-specific distinguishing expression profiles. Only two clusters were clearly annotated: oligodendrocytes and astrocytes (Additional file 1: Figure S3). These results suggest that the expression of these markers, as captured by snuclRNA-seq, is not sufficient to cluster the nuclei by cell type.

### The Consensus Gene Set generates clusters that show cell type-specific expression profiles

Finally, we envisioned an approach to generate data-driven clusters while avoiding biases introduced by overrepresentation and underrepresentation of donors in individual clusters. We explored whether the employment of common genes that are highly variable for each of the three donors would produce viable nuclei clusters (ConGen Methods). We detected 1434 highly variable genes that we used to cluster the nuclei and identified 14 bins (resolution 0.6). The nuclei in each of the clusters were evenly distributed among the three samples with overall high entropy values (Additional file 1: Table S6), and the expression profiles were specific enough to distinguish distinct cell types and subtypes (Additional file 1: Figure S6).

**Fig. 3:**
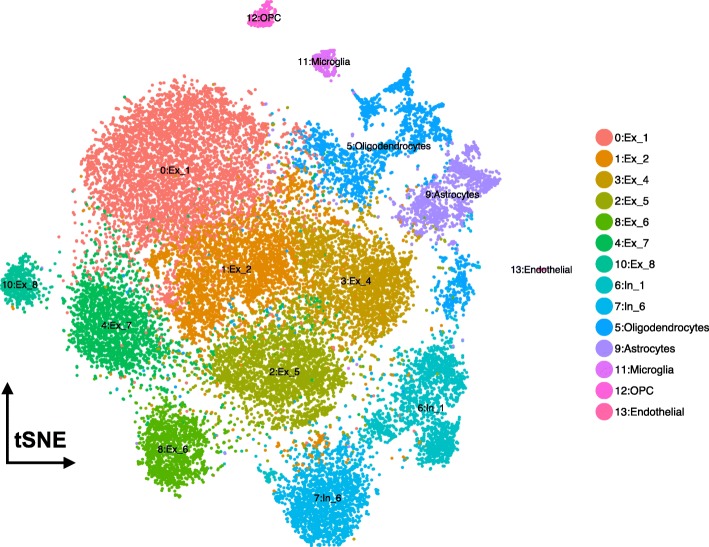
TSNE plots for Consensus Gene Set dimensional reduction approach. TSNE plots depicting 26,331 cells in 14 annotated clusters: Cluster0-Ex_1, Cluster1-Ex_2, Cluster3-Ex_4, Cluster2-Ex_5, Cluster8-Ex_6, Cluster4-Ex_7, Cluster10-Ex_8, Cluster6-In_1, Cluster7-In_6, Cluster5-Oligodendrocytes, Cluster9-Astrocytes, Cluster11-Microglia, Cluster12-OPC, and Cluster13-Endothelial. *In*, inhibitory neuron; *Ex*, excitatory neuron

**Fig. 4:**
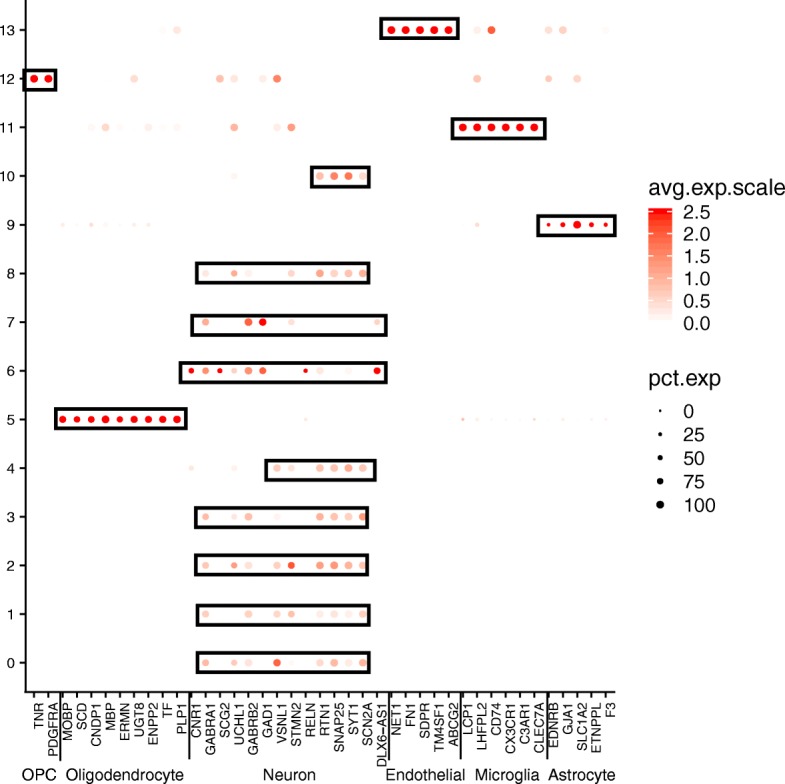
DotPlot depicting the expression of marker genes selected by the literature for the ConGen approach (see Additional file 1: Table S2 and Table S3). This graphical approach allows us to annotate the clusters that were obtained by after the selection of the 1434 common genes

Based on these promising results, we generated gene expression imputed data to reduce the possible technical zeros (dropouts). From this new data, we identified 13 clusters (resolution 0.6, see Table 4). The gene expression patterns specific to cell type clusters were visualized using tSNE plot and DotPlot to represent the expression of gene markers of brain cell types (Fig. 3, Fig. 4). A coincidence analysis identified a broad similarity between the clusters generated from the gene expression imputed data and the non-imputed data (Additional file 1: Figure S4). All of the clusters in the non-imputed data were also identified when we re-analyzed the imputed data, with the exception of two neuronal clusters (7 and 10) that were merged into a single cluster (cluster 6) after gene expression imputation.

**Table 4:** Number of cells for each subject in each cluster using imputed Consensus Gene Set data

				Entropy*
Subject	Sample1	Sample2	Sample3	
Number of total cells	5663	7147	13,521	
Cluster 0	17.36%	27.16%	18.76%	1.56
Cluster 1	18.68%	17.01%	9.99%	1.54
Cluster 2	9.27%	11.70%	10.69%	1.58
Cluster 3	8.83%	3.86%	14.28%	1.41
Cluster 4	9.34%	7.51%	8.65%	1.58
Cluster 5	10.10%	7.43%	6.96%	1.56
Cluster 6	7.77%	8.17%	6.69%	1.58
Cluster 7	6.29%	6.95%	7.49%	1.58
Cluster 8	6.20%	6.91%	3.78%	1.54
Cluster 9	1.08%	0.74%	7.66%	0.89
Cluster 10	2.95%	1.53%	1.96%	1.53
Cluster 11	0.71%	0.53%	1.52%	1.43
Cluster 12	1.32%	0.14%	1.38%	1.23
Cluster 13	0.11%	0.35%	0.18%	1.42

*Entropy values low < 1.2 values indicates uneven sample representation in the cluster

**Fig. 5:**
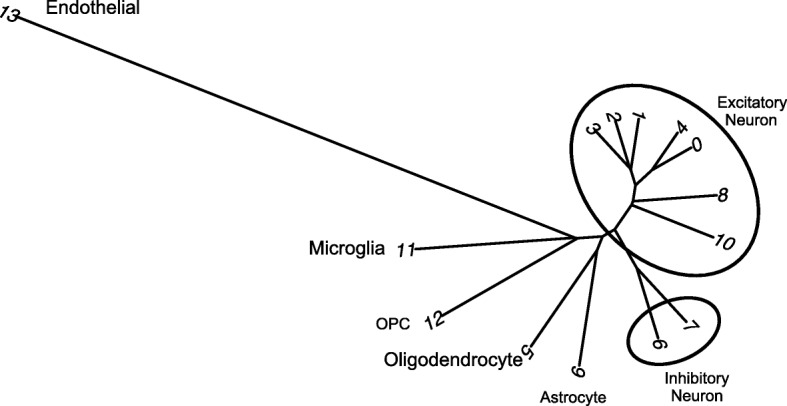
Dendrogram for Consensus Gene Set clusters. This dendrogram shows the hierarchical relationship between clusters, based on the Euclidean distance of cluster mean expression. The proximity of that clusters 0, 1, 2, 3, 4, 8, and 10 indicates the same cell type (excitatory neurons). Inhibitory neurons (clusters 6 and 7) are placed in the same branch as the excitatory neuron. This is another way to confirm the clustering obtained by TSNE

**Fig. 6:**
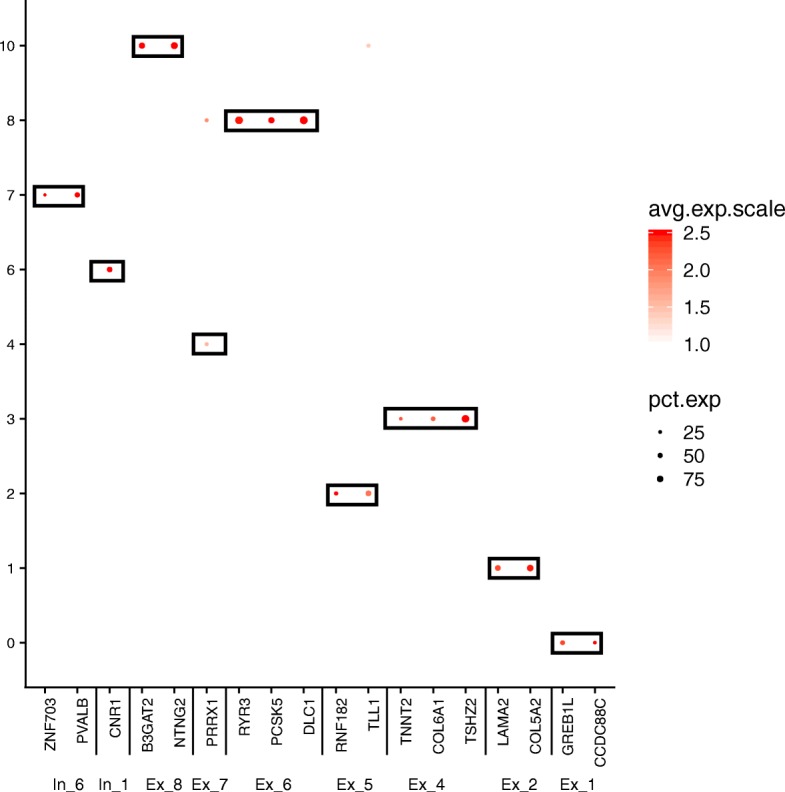
DotPlot depicting the expression of the neuron cell for ConGen approach. Inhibitory neurons are distributed between clusters 6 and 7, and the excitatory neurons are distributed in clusters 0, 1, 2, 3, 4, and 8 as defined by Lake et al. [5]. *In*, inhibitory neuron; *Ex*, excitatory neuron

**Fig. 7:**
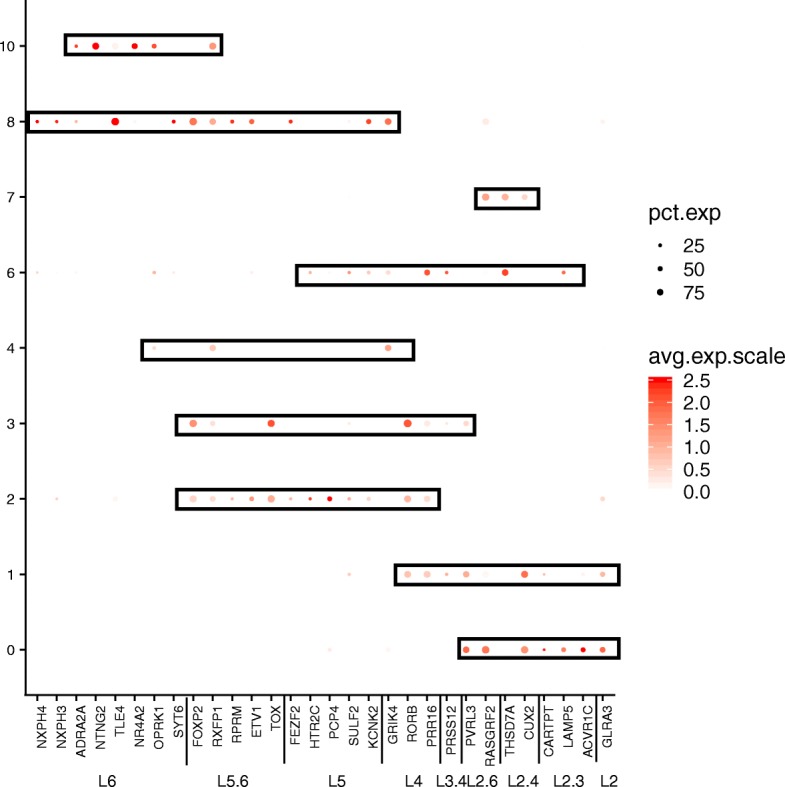
Dot Plots of layer markers in different subclusters of neuron cell subclusters from ConGen approach. DotPlot depicting the expression of layer-specific marker genes going from the superficial layers (e.g., L2) to the deeper layers (L6) for each neural clusters (clusters 0, 1, 2, 3, 4, 6, 7, 8)

Overall, based on the global expression patterns and similarity relationship among the clusters that we inferred (see the “Methods” section), we were able to classify all the cells into three major groups, namely neurons (both excitatory and inhibitory), glial cells (astrocytes, oligodendrocyte, and OPC), and non-neural (endothelial and microglia) (Fig. 5). Lake et al. [5] established a series of neural submarkers that we employed to identify six subclasses of excitatory (Ex) neurons and two subclasses of inhibitory (In) neurons (Fig. 6). The inhibitory subgroups were distributed in different cerebral cortex layers (Fig. 7). Inhibitory cell type 6 (In_6) was located in layer 2. The inhibitory cell type 1 (In_1) was located between layer 2 and layer 5. In case of the excitatory neurons, we observed that most of the subclasses occupied multiple layers (Fig. 7). In more detail, excitatory cell type 1 (Ex_1) covered the whole layer 2 while excitatory cell type 2 (Ex_2) from layer 2 to layer 4. Excitatory cell type 4 (Ex_4), excitatory cell type 5 (Ex_5), and excitatory cell type 6 (Ex_6) spread among layers 4 to 6. Finally, excitatory cell type 8 (Ex_8) covered layers 5 to 6.

All of the clusters, except cluster 9 (Astrocytes), showed an entropy > 1.2 which indicates an even distribution of the nuclei among the samples. Sample3 is overrepresented in cluster 9, as 90% of the cells are from this donor (entropy = 0.89; Additional file 1: Figure S6), but no additional clusters included astrocytes for the other two samples. We evaluated whether our stringent QC process would remove astrocytes for Sample1 and Sample2 and introduce a bias in our data. To do so, we reduced the stringency of the QC parameters and evaluated whether multi-modal distributions were modeling more accurately the number of genes expressed per nuclei, under the assumption that glial cells may express a lower number of genes [11] that would be removed during the cleaning process. However, using this multi-modal distribution (Additional file 1: Figure S8) for QC and cleaning did not recover additional astrocytes nuclei. This suggests that some artifact prevented the efficient capture of astrocytes for these two samples. It has been previously reported that single-cell dissociation, capture, amplification, and sequencing may distort brain cell abundances [11, 37]. In particular, mouse brain stainings were shown not to be correlated perfectly with scRNA-seq, suggesting that neurons were overrepresented relative to non-neurons [11, 37].

We performed a coincidence analysis between the ConGen and CGS approaches (Additional file 1: Figure S6) to qualitatively compare these two approaches. Most of the neural cells from CGS were reorganized into neuronal clusters using the ConGen. This indicates that ConGen is not introducing any novel bias or artifact, and the nuclei are grouped by cell type. Similarly, oligodendrocytes, which were grouped into two clusters by the CGS approach (clusters 6 and 11), were merged into a single cluster in the ConGen. The remaining cells were coincidently grouped into endothelial, oligodendrocyte precursor cell (OPC), microglia, and astrocyte clusters by both approaches. Overall, these results indicated that the ConGen approach clusters cells in a manner that distinguishes the cell type-specific expression profiles that match those generated by the CGS approach but reorganized neurons to avoid underrepresentation or overrepresentation of subjects.

### Single-nuclei RNA-seq reveals specific differences between the brain of the *PSEN1* p.A79V carrier and the two family members with sporadic AD

Neuronal cells accounted for 86.7%, 90.8%, and 82.3% of the cells for Sample1, Sample2, and Sample3, respectively (Table 5). Once we broke down this number based on the neural cell subtype, we observed that the inhibitory neurons for Sample1, Sample2, and Sample3 were 14.1%, 15.2%, and 14.2%, respectively. However, Sample3 (*PSEN1* carrier) showed fewer excitatory neurons (68.1%) compared to Sample1 (72.6%) and Sample2 (75.7%). Overall, this result supports our previous work [4], in which we reported a decreased neuronal percentage (20%) for carriers of pathological mutations in *APP*, *PSEN1*, and *PSEN2* and further suggests that this difference might be specific for excitatory neurons. In addition, we evaluated whether APOE status affects the clustering of the nuclei, but the data suggest that APOE has no effect (Additional file 1: Table S7).

**Table 5:** Number of cells for each sample in each cell type cluster

Subjects			Sample1	Sample2	Sample3
Number of total cells			5663	7147	13,521
Cluster 6	In_1	Inhibitory neurons cells	14.06%	15.18%	14.18%
Cluster 7	In_6				
Cluster 0	Ex_1	Excitatory neurons cells	72.63%	75.68%	68.12%
Cluster 1	Ex_2				
Cluster 3	Ex_4				
Cluster 2	Ex_5				
Cluster 8	Ex_6				
Cluster 4	Ex_7				
Cluster 10	Ex_8				
Cluster 9	Astrocytes	Glial cells	1.08%	0.74%	7.66%
Cluster 5	Oligodendrocytes		10.10%	7.43%	6.96%
Cluster 12	OPC		1.32%	0.14%	1.38%
Cluster 11	Microglia	Non-neural cells	0.71%	0.53%	1.52%
Cluster 13	Endothelial		0.11%	0.35%	0.18%

*In* inhibitory neuron, *Ex* excitatory neuron

### Evaluation of disease-associated microglia marker genes in PSEN p.A79V carrier and sporadic AD

The analyses of sorted microglial cells from mouse models reported a novel microglia type associated with neurodegeneration [10] and the role of *TREM2* in their activation program [38]. This previous study performed immunohistochemical staining of the human brain to identify the colocalization of Aβ particles with *SPP1*, a gene identified as a DAM marker [10]. Since then, much effort has been invested to identify the expression profile of DAM in the human brains, including the analyses of bulk RNA-seq from homogenized human AD brains [38] and bulk RNA-seq from microglia sorted from ten fresh autopsy samples [39]. We hypothesize that the snuclRNA-seq data captures both the expression profiles of DAM cells proximal to Aβ plaques and microglial cells distal to Aβ plaques. We sought to investigate whether the expression profiles of microglial cells we called from the unsorted single-nuclei RNA-seq recapitulate the DAM marker genes. To do so, we performed an in silico pseudo-time reconstruction to capture an ordered sequence of the activation and transition to DAM cells from the transcriptomic profile of microglial cells [22]. From the 500 genes significantly associated with DAM [10] in mice AD models, we identified 326 homolog genes in the human genome. Single-nuclei RNA-seq detected the expression of 92 of these genes in microglial cells (Additional file 1: Table S8). Surprisingly, 79 of these DAM markers show an expression significantly associated with the pseudo-time (*q* value < 0.05). To further investigate whether any artifact was introduced by the possibly distinct expression profiles of microglia from each donor, we performed subject-specific analyses. We determined that only 20 DAM markers were significantly associated with pseudo-time when we analyzed the *PSEN1* carrier (Additional file 1: Table S8). Similarly, 20 and 18 DAM markers were significantly associated with pseudo-time when we analyzed the microglial cells from the sporadic AD brains. Furthermore, five genes were consistently significantly associated with the microglial cells from all three samples, namely *EEF1A1*, *GLUL*, *KIAA1217*, *LDLRAD3*, and *SPP1*. Curiously, *SPP1*, also referred as osteopontin, is the only one of these genes among the top 10 DAM markers identified in mouse AD models and previously employed in immunohistochemical staining of the human brain [10]. Overall, these results indicate that we did not sequence enough microglial cells to replicate the initial DAM marker gene findings. However, the analysis of this microglia suggests promising results. We understand that sorting for glial cells to increase the microglia nuclei count would be optimally posed to provide results that are more definitive.

### A new web-based tool to explore the molecular atlas of Mendelian and sporadic AD brains

Our web-based application (http://ngi.pub/snuclRNA-seq/) provides interactive access to the single-nuclei transcriptomic profiles of the three brains we analyzed. This application is designed to provide a user-friendly and comprehensive analysis of our data, through any modern web browser. The site features a graphical representation (tSEN projection) of the nuclei whose clusters are colored by their cell type, while allowing the selection of the genes with detected expression. For each gene, a graphical representation of its expression profile in the distinct cell types is produced, as well as a statistical interpretation of the differential expression among cell types.

We have regrouped all of the nuclei that were clustered in excitatory and inhibitory clusters that represented neuronal subtypes into a new cluster that represent all of the neurons (Fig. 5). We have precomputed the statistical significance of the differential expression and also the multiple comparisons correction *p* value using Bonferroni multiple test correction. Using this tool, we validated the marker genes that we utilized to annotate the clusters (Additional file 1: Table S2) are significantly overexpressed for the expected cell types. Furthermore, this tool provides a single-nuclei cell type-specific expression reference that we believe will benefit additional research projects, including the annotation of data from other experiments, as well as helping to determine the expression profile of the distinct brain cell types of candidate genes identified in GWAS and sequencing projects.

## Discussion

The complexity and uniqueness of the cell types in the different regions and layers in the human brain make it difficult to understand the implication of each cell type in Alzheimer’s disease. Single-cell RNA-seq is a technology that is maturing rapidly. It is being used to generate a detailed molecular atlas of the brain and explore AD-induced damage at a cellular level. However, this technology is constrained to the analyses of fresh tissue. Single-nuclei RNA sequencing (snuclRNA-seq) is still in its initial developmental stages. In this study, we showed that it is feasible to employ it to ascertain highly informative and unique brain tissue collected during many years and stored in brain banks. We showed that it is possible to ascertain a sufficient and diverse number of transcripts to identify different cell types from three frozen brains from family members with different forms of Alzheimer’s disease. To our knowledge, this is the first study to accomplish this.

Using this data, we generated a highly detailed molecular map of human brains for a carrier of a Mendelian mutation in *PSEN1* and two sporadic ADs. We profiled three major groups of cells in the brain, neurons, glial cells (astrocytes, oligodendrocyte, and OPC), and non-neuronal (endothelial and microglial). Furthermore, we identified distinct types of inhibitory and excitatory neurons. To do so, we extended current de facto methods to correctly process and analyze snuclRNA-seq data and optimize the number of reads, genes, and cells available for downstream analyses.

We showed the limitations and biases introduced by alternative approaches, CGS and Hicat Gene Markers, which were proven to produce reliable results for single-cell RNA-seq. The main problems that we faced were either the uneven representation of subjects in many clusters or clusters with cells that do not show a cell type-specific expression profile. These problems originated from the approach employed to select the genes to cluster the nuclei. Although these methods were effective to cluster cells from single-cell RNA sequencing of fresh tissue or cloned animal models, they were not successful for snuclRNA-seq data.

Our approach called ConGen is based on the identification of a common set of highly variable genes in common among the family-related brains. This allowed us to infer clusters that represent distinct cell types and subtypes, while providing an approximately even representation of cells from all of the subjects in the clusters.

We used this information to analyze the cellular population structure to distinguish structures that are specific to Mendelian AD and differ from sporadic AD. Our results showed a reduced percentage of excitatory neurons in the brain carrier of a *PSEN1* mutation in comparison with two sporadic AD brains. This finding is concordant with our previous observations that Mendelian AD has distinctive and significantly decreased neuronal cell proportions. This result allows us to hypothesize that this phenomenon is specific for excitatory neurons in carriers than sporadic AD, but additional samples should be studied to verify this hypothesis.

We used the microglial cells to study their differential transcriptomic profiles. To our knowledge, this is the first attempt to study DAM markers using unsorted snuclRNA-seq from a *PSEN1 p.A79V* carrier and related AD brains. We showed that snuclRNA-seq provides accurate information, but we understand that the limited number of microglial cells that we sequenced does not provide enough samples to perform unbiased analyses. In addition, our pseudo-temporal ordering analyses of pooled microglia differed from that of donor-specific microglia. We think that these analyses should be performed in additional larger datasets. If any difference among donors is systematically identified, the methods should be extended to correct for any donor-specific effects that might confound the analyses. Still, our analysis replicated the association of osteopontin (*SPI1*) with a temporal trajectory. We believe that by increasing the number of brains and by sorting the nuclei to capture glial cells, we will provide the power required to analyze and detect the expression profile and trajectories of DAM markers in AD brains.

Although all these results are encouraging, we recognize the limitation posed by the small sample size as well as these findings are specific to one carrier *PSEN1 p.A79V*. In addition, we could not identify any family-related neuropathology-free sample to employ as control. While underpowered, it is interesting to note that some of these results replicate the results of previous studies that were designed to provide sufficient statistical power to comparisons.

## Conclusions

**Fig. 8:**
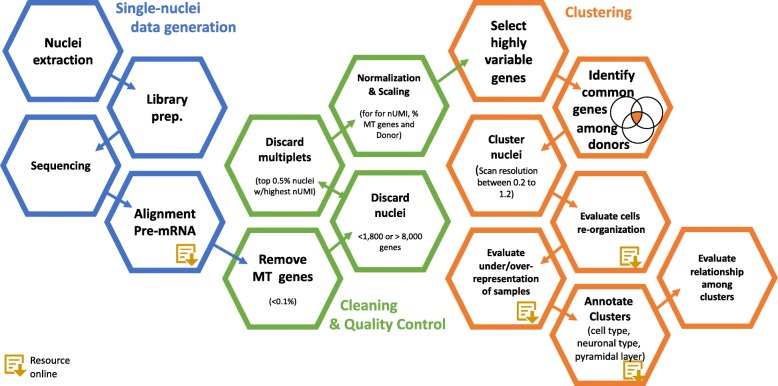
Workflow analysis plan. In blue, the single-nuclei data generation. The most important step is the quantification of the nuclei using a “pre-mRNA” annotation. This step will significantly increase the quantity of nuclei and the quantification of their expression profile. In green, the cleaning and quality control steps. The QC followed standard measurements such as removing mitochondrial genes (MT), removing doublets and multiples, and the normalization of the data using nUMI, percent mitochondrial reads sample origin as confounding factors. In orange, the clustering. In this step, we performed the identification of genes that are highly variable in common among brain nuclei from all of the subjects. Later on, the nuclei can be clustered differently using different resolution, but in general, they are assigned to clusters that are annotated to group nuclei from the same specific cell type. Next, we identified a hierarchical relationship among the clusters by performing coincidence analyses. The entropy, from Shannon’s information theory, provides a quantitative measure of even distribution of samples in a cluster. To annotate the clusters, we use a set of gene markers for each cell type collected from the literature. Finally, a hierarchical clustering of clusters should reproduce expected results, grouping together neuronal subtypes in one branch and in another branch glial cells

Overall, we believe that this work proposes best practices for the generation, processing, and analyses of single-nuclei RNA-seq (Fig. 8) data that maximizes the amount of information able to be extracted from the samples. These are the lessons we learned while analyzing these brains: (i) The quantification of the nuclei using a “pre-mRNA” annotation will significantly increase the quantity of nuclei and the quantification of their expression profile. (ii) Identification of genes that are highly variable in common among the brain nuclei produce clusters with an even representation of all of the subjects. (iii) Nuclei can be clustered differently using different resolution, but in general, they are assigned to clusters that are annotated to the group nuclei from the same specific cell type. In addition, we identified a hierarchical relationship among the clusters as a product of different approaches or levels of resolution. To reveal these relationships, we propose coincidence analyses. (iv) Shannon’s information theory of entropy should be as a quantitative measure of even distribution of all samples in all clusters. (v) To annotate each cluster, we need to use a consensus set of gene markers for each cell type from the current literature. There is not a standard set of gene markers. (vi) A hierarchical clustering of clusters should reproduce expected results, grouping together neuronal subtypes in one branch and in another branch glial cells.

Finally, we have generated a highly detailed molecular atlas of AD brains that we are making available through an interactive, user-friendly, web-based application. We believe it will help not only the annotation of other snuclRNA-seq studies, but also additional high-throughput neurodegenerative genomic studies.

## Additional file

Additional file 1: **Table S1.** 118 cell type markers for Hicat Gene Marker. **Table S2.** Cell type markers. **Table S3.** Cerebral cortex layer-specific markers. **Table S4.** Distribution of nuclei per subject using CGS. **Table S5.** Distribution of nuclei per subject using Hicat Marker Gene. **Table S6.** Distribution of nuclei per subject using Consensus Gene Set. **Table S7.** Number of cells for Sample1 and Sample2 in each cluster using imputed Consensus Gene Set data. **Table S8.** DAM markers significantly associated with pseudo-time. **Figure S1.** Single nuclei after extraction from sample1. **Figure S2.** DotPlot depicting the expression of marker genes selected from the literature (see Table S2 and Table S3) on Classic Gene Set data. **Figure S3.** Hicat Gene Markers Dimensional Reduction approach. **Figure S4.** DotPlot depicting the expression of marker genes. **Figure S5.** Coincidence analysis between Consensus Gene Set and its gene expression imputed counterpart. **Figure S6.** Coincidence analysis between Classic Gene Set and its Consensus Gene Set. **Figure S7.** Entropy data. **Figure S8.** Multi-modal distributions of unique molecular identifier (UMI) per nuclei. (DOCX 4806 kb)

## Abbreviations

AD: Alzheimer’s disease
APP: Amyloid-beta precursor protein
CDR: Clinical Dementia Rating
CGS: Classic Gene Set
ConGen: Consensus Gene Set
DAM: Disease-associated microglia
Ex: Excitatory neurons cell type
GWAS: Genome-wide association study
In: Inhibitory neurons cell type
Knight-ADRC: Knight-Alzheimer’s Disease Research Center
mRNA: Mature messenger RNA
NIA-AA: National Institute on Aging-Alzheimer’s Association
nUMI: Number of unique molecular identifiers
OPC: Oligodendrocyte precursor cells
PC: Principal components
pmi: Pointwise mutual information
PMI: Postmortem interval
pre-mRNA: Nuclear precursor mRNA
PSEN1: Presenilin 1
PSEN2: Presenilin 2
scRNA-seq: Single-cell RNA-seq
snuclRNA-seq: Single-nuclei RNA-seq
tSNE: T-distributed Stochastic Neighbor Embedding

## References

[1] LaFerla FM, Oddo S (2005). Alzheimer’s disease: Aβ, tau and synaptic dysfunction. Trends Mol Med.

[2] De Strooper B, Annaert W (2010). Novel research horizons for presenilins and gamma-secretases in cell biology and disease. Annu Rev Cell Dev Biol.

[3] Selkoe DJ (2001). Alzheimer’s disease: genes, proteins, and therapy. Physiol Rev.

[4] Li Z, Del-Aguila JL, Dube U, Budde J, Martinez R, Black K (2018). Genetic variants associated with Alzheimer’s disease confer different cerebral cortex cell-type population structure. Genome Med.

[5] Lake BB, Ai R, Kaeser GE, Salathia NS, Yung YC, Liu R (2016). Neuronal subtypes and diversity revealed by single-nucleus RNA sequencing of the human brain. Science..

[6] 10XGenomics. Genomics X: isolation of nuclei for single cell RNA sequencing.

[7] Qiu X, Mao Q, Tang Y, Wang L, Chawla R, Pliner HA (2017). Reversed graph embedding resolves complex single-cell trajectories. Nat Methods.

[8] Zheng GX, Terry JM, Belgrader P, Ryvkin P, Bent ZW, Wilson R (2017). Massively parallel digital transcriptional profiling of single cells. Nat Commun.

[9] Grindberg RV, Yee-Greenbaum JL, McConnell MJ, Novotny M, O’Shaughnessy AL, Lambert GM (2013). RNA-sequencing from single nuclei. Proc Natl Acad Sci U S A.

[10] Genomics Creating a Reference Package with cellranger mkref. https://support.10xgenomics.com/single-cell-gene-expression/software/pipelines/latest/advanced/references#header. Accessed 10 July 2019

[11] Saunders Arpiar, Macosko Evan Z., Wysoker Alec, Goldman Melissa, Krienen Fenna M., de Rivera Heather, Bien Elizabeth, Baum Matthew, Bortolin Laura, Wang Shuyu, Goeva Aleksandrina, Nemesh James, Kamitaki Nolan, Brumbaugh Sara, Kulp David, McCarroll Steven A. (2018). Molecular Diversity and Specializations among the Cells of the Adult Mouse Brain. Cell.

[12] Benaglia T, Chauveau D, Hunter DR, Young DS. mixtools: An R package for analyzing mixture models. J Stat Softw. 2010;32(6). 10.18637/jss.v0.32.i06. https://www.jstatsoft.org/article/view/v032i06.

[13] Xu C, Su Z (2015). Identification of cell types from single-cell transcriptomes using a novel clustering method. Bioinformatics..

[14] Chen R, Wu X, Jiang L, Zhang Y (2017). Single-cell RNA-Seq reveals hypothalamic cell diversity. Cell Rep.

[15] Hu P, Fabyanic E, Kwon DY, Tang S, Zhou Z, Wu H (2017). Dissecting cell-type composition and activity-dependent transcriptional state in mammalian brains by massively parallel single-nucleus RNA-Seq. Mol Cell.

[16] Tasic B, Yao Z, Graybuck LT, Smith KA, Nguyen TN, Bertagnolli D (2018). Shared and distinct transcriptomic cell types across neocortical areas. Nature..

[17] ABCAM. Neural Lineage MArkers at a Glance. https://www.abcam.com/neuroscience/neural-markers-guide. Accessed 10 July 2019.

[18] McKenzie AT, Wang M, Hauberg ME, Fullard JF, Kozlenkov A, Keenan A (2018). Brain cell type specific gene expression and co-expression network architectures. Sci Rep.

[19] Rice Heather C., de Malmazet Daniel, Schreurs An, Frere Samuel, Van Molle Inge, Volkov Alexander N., Creemers Eline, Vertkin Irena, Nys Julie, Ranaivoson Fanomezana M., Comoletti Davide, Savas Jeffrey N., Remaut Han, Balschun Detlef, Wierda Keimpe D., Slutsky Inna, Farrow Karl, De Strooper Bart, de Wit Joris (2019). Secreted amyloid-β precursor protein functions as a GABABR1a ligand to modulate synaptic transmission. Science.

[20] Li WV, Li JJ (2018). An accurate and robust imputation method scImpute for single-cell RNA-seq data. Nat Commun.

[21] Shannon CE (1997). The mathematical theory of communication. 1963. MD Comput.

[22] Ji Z, Ji H (2016). TSCAN: pseudo-time reconstruction and evaluation in single-cell RNA-seq analysis. Nucleic Acids Res.

[23] Gandal MJ, Haney JR, Parikshak NN, Leppa V, Ramaswami G, Hartl C (2018). Shared molecular neuropathology across major psychiatric disorders parallels polygenic overlap. Science..

[24] Jaffe AE, Shin J, Collado-Torres L, Leek JT, Tao R, Li C (2015). Developmental regulation of human cortex transcription and its clinical relevance at single base resolution. Nat Neurosci.

[25] Kelley KW, Nakao-Inoue H, Molofsky AV, Oldham MC (2018). Variation among intact tissue samples reveals the core transcriptional features of human CNS cell classes. Nat Neurosci.

[26] Oldham MC, Konopka G, Iwamoto K, Langfelder P, Kato T, Horvath S (2008). Functional organization of the transcriptome in human brain. Nat Neurosci.

[27] Voineagu I, Wang X, Johnston P, Lowe JK, Tian Y, Horvath S (2011). Transcriptomic analysis of autistic brain reveals convergent molecular pathology. Nature..

[28] Wang Daifeng, Liu Shuang, Warrell Jonathan, Won Hyejung, Shi Xu, Navarro Fabio C. P., Clarke Declan, Gu Mengting, Emani Prashant, Yang Yucheng T., Xu Min, Gandal Michael J., Lou Shaoke, Zhang Jing, Park Jonathan J., Yan Chengfei, Rhie Suhn Kyong, Manakongtreecheep Kasidet, Zhou Holly, Nathan Aparna, Peters Mette, Mattei Eugenio, Fitzgerald Dominic, Brunetti Tonya, Moore Jill, Jiang Yan, Girdhar Kiran, Hoffman Gabriel E., Kalayci Selim, Gümüş Zeynep H., Crawford Gregory E., Roussos Panos, Akbarian Schahram, Jaffe Andrew E., White Kevin P., Weng Zhiping, Sestan Nenad, Geschwind Daniel H., Knowles James A., Gerstein Mark B. (2018). Comprehensive functional genomic resource and integrative model for the human brain. Science.

[29] Bruner E, Jacobs HI (2013). Alzheimer’s disease: the downside of a highly evolved parietal lobe?. J Alzheimers Dis.

[30] Cabeza R, Ciaramelli E, Olson IR, Moscovitch M (2008). The parietal cortex and episodic memory: an attentional account. Nat Rev Neurosci.

[31] Jacobs HI, Van Boxtel MP, Jolles J, Verhey FR, Uylings HB (2012). Parietal cortex matters in Alzheimer’s disease: an overview of structural, functional and metabolic findings. Neurosci Biobehav Rev.

[32] Lindeboom J, Weinstein H (2004). Neuropsychology of cognitive ageing, minimal cognitive impairment, Alzheimer’s disease, and vascular cognitive impairment. Eur J Pharmacol.

[33] Bakken TE, Hodge RD, Miller JA, Yao Z, Nguyen TN, Aevermann B (2018). Single-nucleus and single-cell transcriptomes compared in matched cortical cell types. PLoS One.

[34] Lake BB, Chen S, Sos BC, Fan J, Kaeser GE, Yung YC (2018). Integrative single-cell analysis of transcriptional and epigenetic states in the human adult brain. Nat Biotechnol.

[35] Wang J, Chen L, Chen Z, Zhang W (2015). RNA-seq based transcriptomic analysis of single bacterial cells. Integr Biol (Camb).

[36] Butler A, Hoffman P, Smibert P, Papalexi E, Satija R (2018). Integrating single-cell transcriptomic data across different conditions, technologies, and species. Nat Biotechnol.

[37] Herculano-Houzel S (2014). The glia/neuron ratio: how it varies uniformly across brain structures and species and what that means for brain physiology and evolution. Glia..

[38] Friedman BA, Srinivasan K, Ayalon G, Meilandt WJ, Lin H, Huntley MA (2018). Diverse brain myeloid expression profiles reveal distinct microglial activation states and aspects of Alzheimer’s disease not evident in mouse models. Cell Rep.

[39] Olah M, Patrick E, Villani AC, Xu J, White CC, Ryan KJ (2018). A transcriptomic atlas of aged human microglia. Nat Commun.

